# Efficient Binding of the NOS1AP C-Terminus to the nNOS PDZ Pocket Requires the Concerted Action of the PDZ Ligand Motif, the Internal ExF Site and Structural Integrity of an Independent Element

**DOI:** 10.3389/fnmol.2017.00058

**Published:** 2017-03-15

**Authors:** Li-Li Li, Katryna Cisek, Michael J. Courtney

**Affiliations:** ^1^Molecular Signalling Laboratory, Department of Neurobiology, A. I. Virtanen Institute, University of Eastern FinlandKuopio, Finland; ^2^Neuronal Signalling Laboratory, Turku Centre for Biotechnology, University of TurkuTurku, Finland

**Keywords:** nos1ap, nNOS, PDZ domain, fluorescence polarization, dissociation constant, molecular dynamics simulation, multi-site binding, ZLc-002-1

## Abstract

Neuronal nitric oxide synthase is widely regarded as an important contributor to a number of disorders of excitable tissues. Recently the adaptor protein NOS1AP has emerged as a contributor to several nNOS-linked conditions. As a consequence, the unexpectedly complex mechanisms of interaction between nNOS and its effector NOS1AP have become a particularly interesting topic from the point of view of both basic research and the potential for therapeutic applications. Here we demonstrate that the concerted action of two previously described motif regions contributing to the interaction of nNOS with NOS1AP, the ExF region and the PDZ ligand motif, efficiently excludes an alternate ligand from the nNOS-PDZ ligand-binding pocket. Moreover, we identify an additional element with a denaturable structure that contributes to interaction of NOS1AP with nNOS. Denaturation does not affect the functions of the individual motifs and results in a relatively mild drop, ∼3-fold, of overall binding affinity of the C-terminal region of NOS1AP for nNOS. However, denaturation selectively prevents the concerted action of the two motifs that normally results in efficient occlusion of the PDZ ligand-binding pocket, and results in 30-fold reduction of competition between NOS1AP and an alternate PDZ ligand.

## Introduction

NMDA receptor mediated signaling through nNOS is increasingly recognized as a contributor to a number of neurological conditions including stroke, depression and neuropathic pain (Florio et al., 2009; Hill et al., 2012; Doucet et al., 2013; Lee et al., 2015). Experimental models suggest that NOS1AP may mediate the actions of NMDA-driven nNOS function for example in excitotoxic conditions such as neonatal hypoxia and anxiety induced by chronic mild stress (Li et al., 2013; Zhu et al., 2014). It has been specifically suggested that nNOS-NOS1AP interaction could be a therapeutic target in schizophrenia (Weber et al., 2014; Freudenberg et al., 2015), while NOS1AP has emerged as a highly significant associated gene in cardiovascular conditions (Arking et al., 2006; Kapoor et al., 2014). For these reasons the interaction between nNOS and NOS1AP is emerging as a potential target for drug development. Both peptide and small molecule inhibitors of the interaction have already been reported (Li et al., 2013, 2015; Zhu et al., 2014), and improved understanding of the interaction between nNOS and NOS1AP may assist in future drug development.

The interaction had originally been considered to be a canonical PDZ interaction (Jaffrey et al., 1998; discussed in Courtney et al., 2014), which involves the C-terminal 7–9 residues of a ligand (NOS1AP in this case) docking into a well-defined pocket in a PDZ domain (between residues 14–98 of nNOS in this case, Tochio et al., 1999). Supporting this, deletion of the C-terminus of NOS1AP eliminates binding to nNOS by conventional pulldown or immunoprecipitation methods (Jaffrey et al., 1998; Li et al., 2015; Candemir et al., 2016). Conflicting with this, is that the last 9–20 residues of NOS1AP are not sufficient for interaction to be detected by either pulldown or immunoprecipitation methods (Li et al., 2015; Candemir et al., 2016). The explanation for this finding is that the C-terminal PDZ ligand motif alone has exceedingly low affinity for nNOS (Li et al., 2015) but it nevertheless contributes stabilization to a primary interaction with an internal NOS1AP sequence containing an ExF motif. This results in ∼5-fold increased affinity and a ∼5-fold slower off-rate and, in the context of multiple wash steps of a conventional pulldown assay this amounts to the difference between strong binding and no binding (Li et al., 2015).

Thus the concerted action of these two interaction sites leads to a longer-lived protein complex and this appears to be important for the efficiency of mediation of signal transduction pathways, specifically the activation of the p38 pathway and excitotoxic neuronal cell death (Li et al., 2015). Nevertheless, it has not been shown whether docking of NOS1AP to nNOS via the ExF motif alone has any influence on the competence of the nNOS PDZ pocket to bind ligands, nor even the apparent affinity with which NOS1AP occupies the actual PDZ ligand-binding pocket when NOS1AP is bound to nNOS. The independence and interdependence of the two nNOS-interacting motifs has not been directly addressed.

Here we report that denaturation of the C-terminal nNOS-binding region (which contains both the ExF motif and the PDZ ligand) does not prevent interaction via the ExF motif, suggesting the latter does not require a structure that can be denatured. Surprisingly, however, we find the denatured binding region interacts with nNOS N-terminus at a similar affinity whether or not the PDZ ligand is present, in sharp contrast to previous findings using the *native* binding region (Li et al., 2015) in which ExF and PDZ ligand regions cooperate to generate high-affinity stable binding of nNOS. As a result, the reported increase in affinity of the C-terminal NOS1AP region containing both nNOS binding motifs is largely lost in the denatured peptide. This suggests PDZ pocket occupancy depends on a native structure. Indeed we show directly that, whereas the two-site binding region of NOS1AP (residues 400–506, human numbering) in native form exhibits high affinity competition against exogenous ligands for the nNOS PDZ-binding pocket (∼0.8 μM), denaturation of this NOS1AP fragment reduces competition at the pocket 30-fold. This shows that a relatively small change in overall affinity of the two-site binding region, NOS1AP[400–506], can mask a considerable drop in binding of one of the sites, and demonstrates the importance of investigating interactions at each site independently where feasible. The PDZ-binding C-terminal ligand of NOS1AP is a short peptide, and a molecular dynamic simulation suggest that no rigid structure is required for docking of the PDZ ligand to nNOS, which appears to only involve the last 3–4 amino acids. However, secondary structure prediction algorithms reveal potential structural elements that form between the ExF motif region and the PDZ ligand. Our data is consistent with a requirement for a structural element outside the ExF and PDZ motifs to be in a native conformation to allow concerted action of the ExF motif and PDZ ligand interactions. In denatured state, even if the two interaction sites can bind nNOS independently of one another, they cannot cooperate with one another to generate the increased apparent affinity of interaction otherwise seen, and this is most likely for steric reasons. The potential relevance of this additional requirement for nNOS-NOS1AP to the development of inhibitory strategies is discussed.

## Materials and Methods

### Peptides

Peptide “GDLV” refers to NH2-RRRRWGDLV-COOH, whereas F-GDLV refers to the N-terminally fluoresceinated variant. Both were obtained from Genic Bio (Shanghai, China) and were of >92 and >97% purity respectively. Peptide “EIAV” refers to NH2-DSLDDEIAV-COOH, corresponding to the last 9 amino acids of rat/mouse NOS1AP, was synthesized as described Li et al. (2013) by Xigen AG (Lausanne, Switzerland). The corresponding human sequence is NH2-DGLDDEIAV-COOH, i.e., a S > G substitution at position -7 from the C-terminal valine. We previously aligned the C-terminal region from multiple species (Li et al., 2015) which showed that birds, reptiles and terrestrial and marine mammals typically have a serine at this position, fish typically have a cysteine, while the primates we aligned have a glycine. As we do not find evidence for the contribution of the residues distal to the C-terminus to binding, and this sequence alone binds nNOS extremely weakly (Li et al., 2015), serine does not appear to contribute to binding. As glycine has no side chain (only two hydrogens on the alpha-carbon), its contribution to binding is unlikely to be greater than serine. Notably, rat, mouse and human nNOS-PDZ domains are identical in the region from residues 6–126 except for D/G substitution at amino acid 69. This residue is not close to the PDZ-ligand binding pocket and faces solvent in solved structures 1B8Q (Tochio et al., 1999) and 1QAV/1QAU (Hillier et al., 1999).

### Antibodies

Antibody against NOS1AP (rabbit polyclonal, R-300, sc-9138, RRID:AB_2251417) was from Santa Cruz Biotech, and Dy-Light coupled secondary antibody was from Cell Signaling Technologies.

### Plasmid constructs

pET28a-TAT-NOS1AP[400–506], pET28a-TAT-NOS1AP[400–503], pET28a-TAT-NOS1AP[400–503] E429A, pET28a-TAT-NOS1AP[400–503] F431A, pET28a-nNOS[1–155], pGEX-nNOS[1–155], pGEX-6P-NOS1AP[400–503], pGEX-6P-NOS1AP[400–506], pGEX-6P-NOS1AP[432–506], pGEX-6P-NOS1AP[400–503] F431A and pGEX-6P-NOS1AP[400–506] F431A, encoding human protein sequences (amino acids as specified) fused to GST, His and His-TAT tags, and pGEX-HRV3C protease expression vector were previously described Li et al. (2015).

### Recombinant Protein Expression and Purification

His-TAT-NOS1AP fusions were purified under native conditions using lysozyme-based lysis conditions (Courtney and Coffey, 1999) or under denaturing conditions as described Becker-Hapak et al. (2001). His-nNOS(1–155) was generated as described Li et al. (2015) as was GST-nNOS(1–155) and GST. All His-nNOS[1–155] used for fluorescence polarization (FP) assays was polished by Superdex200 size-exclusion chromatography as described Li et al. (2015). NOS1AP[400–506], NOS1AP[400–503], NOS1AP[432–503] and NOS1AP[400–506] F431A were obtained from GST fused versions by on-column cleavage using GST-HRV3C protease as described Li et al. (2015).

### Solid-Phase No-Wash Binding Assay

This was carried out as previously described Li et al. (2015). Briefly, 5 μl beads bound to 1 μg GST-fused nNOS protein (or GST for background measurement) were rotated with concentrations of recombinant His-TAT-NOS1AP peptides as shown for 1 h at 4°C and transferred to multiscreen filter plates (Millipore). Unbound peptides were removed by centrifugation, beads were resuspended in LSB (Cao et al., 2005) and protein eluted with Laemmli buffer. The affinity of each peptide was determined using fluorescent immunoblotting quantified with the Odyssey infrared imaging system (LI-COR) and ImageJ as follows. Bound peptide, together with the background binding of each TAT peptide to GST beads, was quantified in each blot using a set of standard concentrations of the same TAT fragment on the same blot. Background binding to GST, barely detected except at the highest fragment concentration, was subtracted in each case. Fitting was performed for each replicate with Excel using the quadratic solution formula to account for bound ligand, fb = B/L_0_ = {+(K_d_ + P_0_ + L_0_)-√ [(K_d_ + P_0_ + L_0_)^2^ – 4L_0_P_0_]}/2L_0_.

### Fluorescence Polarization and Competition Fluorescence Polarization

FP assays were carried using a BMG Polarstar OPTIMA reader as previously described Li et al. (2015). Briefly, synthetic peptide corresponding to the optimal nonamer ligand for the PDZ ligand-binding pocket of nNOS, based on the peptide scan of Stricker et al. (1997), was labeled with fluorescein at the N-terminus and used as a fluorescent ligand. This is referred to as F-GDLV. Fluorescence titration was performed by adding increasing amounts of His-tagged nNOS(1–155) to a constant amount of the fluoresceinated peptide (1 μM) in FP buffer (50 mM Tris buffer, pH 7.4, containing 100 mM NaCl, 1 mM EDTA, 0.1% BSA). The dissociation constant (K_d_) was obtained by fitting the titration curves with the classical one-site binding model with MS Excel Solver the quadratic solution formula to account for bound ligand, as shown above in the section “solid-phase no wash assay.” The K_d_ values were obtained in triplicate from each of two different batches of His-nNOS, curve fitting was performed on each replicate. The mean ± SEM shown in **Figure 2** and **Table 2** (10.7 ± 0.8 μM) corresponds to the results of curve fittings to the 6 replicates. The individual nNOS batches gave values of 10.2 ± 1.7 μM and 11.2 ± 0.2 μM.

Competition FP was carried out using 1 μM F-GDLV peptide 4 μM His-nNOS(1–155) and increasing amounts of competitor peptide as indicated. Approximate values for Kc, the dissociation constant of the competitor, were obtained using the formula for single site competition as described in Harris et al. (2001). For each experiment the data were fitted with MS Excel Solver according to the measured dissociation constant for the specific nNOS batch used in the experiment (see above).

### Molecular Dynamics Simulation

All simulations were carried out using the Desmond Molecular Dynamics System v2.2 (D. E. Shaw Research, New York, NY, USA) and Schrodinger suite tools (Schrodinger, LLC, Portland, OR, USA) on the supercomputing clusters of the Centre for Scientific Computing (Espoo, Finland). NMR structures (pdb ID 1B8Q) were imported using Schrodinger’s Maestro to assign proper bond orders. Next, the structures were prepared using Schrodinger’s Protein Preparation Wizard, following a standard solvation box with 10 Å buffering, 150 mM Na^+^ and Cl^-^ ion buffering, and SPC water model. The OPLS-AA/2005 force field was used for all simulations with a multi-step minimization procedure using default settings to relax the system prior to simulation. For the molecular dynamics production runs, the NPT ensemble was used and a trajectory for 10 ns generated and visualized using the Maestro Trajectory Player. Three poses are shown at 0.15 ns intervals to show the rapid movement of the non-docked ligand residues compared with the docked ones.

## Results

We previously demonstrated that it is possible to use the ExF site-bearing peptides alone as neuroprotectants in models of excitotoxic neuronal stress. This was achieved using recombinantly expressed and purified TAT-fused peptides that pass the plasma membrane (Li et al., 2015). In this previous report, we found using a quantitative solid-phase binding assay that TAT-fused peptides of NOS1AP (incorporating residues from 400 onward, human usage) exhibited ∼5-fold lower affinity when the PDZ ligand (residues 504–506) was lacking (**Table 1**, column 1; Li et al., 2015). This was consistent with a ∼5-fold lowering of the off-rate of interaction of the nNOS-NOS1AP complex when the PDZ ligand motif was present that we observed using an independent assay system. Additional quantitative and qualitative protein interactions supported this interpretation and suggested that the PDZ motif, while having little affinity itself, nevertheless contributed to the formation of a stable interaction of nNOS with NOS1AP (Li et al., 2015).

**Table 1:** Comparison of K_d_ values for native and denatured forms of NOS1AP for nNOS[1–155] previously reported and shown here.

N0S1AP residues	Native (Li et al., 2015)	Denatured (Figure 1)
400–506	0.75 ± 0.1 μM	2.4 ± 0.3 μM
400–503	5.8 ± 1.0 μM	5.3 ± 0.9 μM
400–503 E429A	1.1 ± 0.3 mM	99 ± 19 μM
400–503 F431A	1.1 ± 0.2 mM	100 ± 20 μM

**FIGURE 1:**
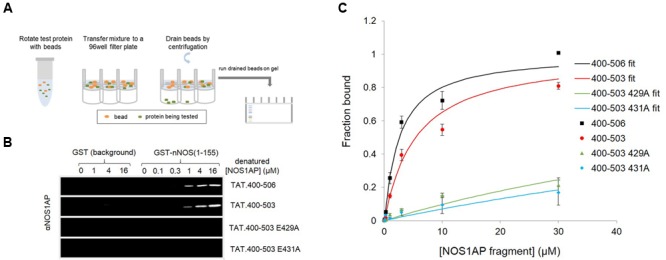
**A native conformation of NOS1AP is required for the concerted action of the PDZ ligand and ExF motif region on binding nNOS in a solid phase no-wash binding assay. (A)** Scheme depicting the no-wash solid-phase binding assay used to measure the interaction of NOS1AP peptides with nNOS. **(B)** Representative fluorescent immunoblot images showing binding of His-TAT-NOS1AP to GST (background binding, left lanes, hardly visible) and GST-nNOS[1–155] (right lanes) at concentrations of protein indicated. Bound peptide was detected by NOS1AP primary antibody and an Odyssey infrared imaging system. **(C)** Curves of specific NOS1AP binding, after correcting for background and normalizing to internal standards on each gel for each protein, from replicates of experiments shown in **(B)**. Data are mean ± SEM (*n* = 3) and superposed curves were fitted to data according to a 1-site model as described in Section “Materials and Methods.”

*Affinity constants were obtained by fitting each replicate for each peptide. Means ± SEM K_*d*_ values are indicated. Native His-TAT protein data is taken from Li et al., 2015 whereas the denatured protein data carried out in parallel is shown here in **Figure 1**. Note that denaturation selectively reduces the affinity of NOS1AP[400–506] ∼3-fold. The increased affinity of the essentially non-binding ExF point mutants lacking of PDZ ligand is an expected consequence of denaturation, but these proteins still have very low affinity for nNOS and this effect is unlikely to influence the specific binding of NOS1AP fragments with functional ExF motifs.*

### A Native Conformation of NOS1AP is Required for the Concerted Action of the PDZ Ligand and ExF Motif Region in Binding nNOS

It is now well established that regions of proteins, domains of proteins and even entire proteins can exist in natively unfolded or intrinsically unstructured states (called IDP or intrinsically disordered proteins; for review see Latysheva et al., 2015; Wright and Dyson, 2015). It is thought that this allows these proteins to adopt confirmations required for target-binding without imposing such conformational constraints in the absence of binding, thereby permitting the binding to different targets. These regions and proteins are resistant to denaturation. In contrast, other regions or often entire proteins are structured. In this case, they form specific structures, and these are denaturable by heat, chemical denaturants or other conditions to which the proteins are not adapted. Thus, the comparison of protein function before and after denaturation is an investigative approach that can be used to determine the importance of structural elements for the specific functions of a protein. This does not necessarily mean that denaturation or loss of structure (or the opposite) is a physiological mechanism of regulation, although in some cases this is case (reviewed in Mitrea and Kriwacki, 2013; Csizmok et al., 2016). The light-induced switch of the J-alpha sequence of plant photoprotein Lov2 domains from alpha-helical to unfolded state (Harper et al., 2003) is one prominent example, which has been exploited by synthetic biologists to generate optogenetic regulators of cell signaling (Strickland et al., 2008; Wu et al., 2009; Lungu et al., 2012; Melero-Fernandez de Mera et al., 2013, 2017; Guntas et al., 2015).

We previously reported two-site affinity measurements for NOS1AP-nNOS interaction using TAT-fused NOS1AP protein expressed and isolated from bacterial expression systems under *native* conditions (Li et al., 2015). Uptake of recombinant TAT-fusions into cells is promoted by denaturation and they are presumed to be refolded in the cytoplasm by intracellular chaperone systems (Becker-Hapak et al., 2001). Considering the prevalence of functional intrinsically disordered regions in the proteome and the lack of a predicted structured domain in the C-terminal region of NOS1AP, we decided to measure affinities of the denatured TAT-proteins in parallel with the native versions, for the same sites using the same no-wash pulldown assay as we previously used for the native proteins (Li et al., 2015). We intentionally used a no-wash pulldown assay in this study because we previously reported that the conventional washing steps, which have no physiological counterpart, greatly exaggerate the impact of PDZ motif on overall binding affinity (Li et al., 2015). **Figure 1** shows that the affinity of nNOS for denatured NOS1AP[400–503], the NOS1AP form that lacks the PDZ ligand motif, is similar to that of the native protein (Li et al., 2015; **Table 1**). However, in contrast to the results obtained with native protein, the affinity difference between NOS1AP[400–506] and NOS1AP[400–503] was almost completely lost, both having a similar affinity to that previously described for native NOS1AP[400–503] (**Table 1**). This suggests that, although the interaction of the internal ExF motif region (present in NOS1AP[400–503]) with nNOS does not require a pre-existing natively folded conformation, the increased affinity obtained by concerted action of the two interaction motifs in NOS1AP[400–506], in contrast *does* require a native structure.

### Fluorescence Polarization Can Be Used to Directly Probe the Occupancy of the PDZ Ligand-Binding Pocket of nNOS

**FIGURE 2:**
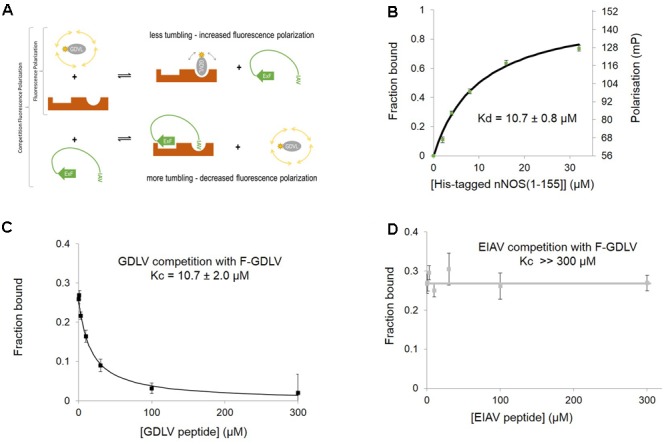
**Fluorescent peptide FITC-RRRRWGDLV can be used as a fluorescence polarization ligand to directly probe the occupancy of the PDZ ligand-binding pocket of nNOS. (A)** Scheme depicting the competition fluorescence polarization (FP) assay. Optimal peptide ligand RRRRWGDLV (shown as gray oval) was N-terminally fluoresceinated and used as an FP probe with acceptable dynamic range and affinity. The PDZ ligand binding pocket of nNOS (Tochio et al., 1999, here shown in brown with two distinct binding sites) can bind to the FP probe (upper right panel) or the IAV motif of NOS1AP which also docks via ExF motif to a second site on nNOS (lower right panel, Li et al., 2015). Thus competition FP reveals binding of peptides such as NOS1AP to the PDZ pocket and its possible facilitation by additional sites such as the ExF motif. We indicate the ExF motif schematically as a β-sheet as secondary structure prediction algorithms suggest it may form this motif (Li et al., 2015) even though our results here suggest the function of this region is resistant to denaturation (**Figure 1**). **(B)** Increasing His-nNOS[1–155] in the presence of F-GDLV ligand (1 μM) reveals FP data fitting a single-site binding curve, based on the free concentration of ligand. A representative curve is shown (*n* = 3), and mean of experiments with two different nNOS batches gave a K_d_ of 10.7 ± 0.8 μM (mean ± SEM, using *n* = 6 curve fittings). **(C)** Unlabelled GDLV peptide competes with F-GDLV (1 μM) for binding His-nNOS[1–155] (4 μM) resulting in a competition curve indicating a Kc (affinity of competitor) of 10.7 ± 2.0 μM (datapoints shown are mean ± SEM, *n* = 3), consistent with the F-GDLV-nNOS binding data in **(B)**. **(D)** Unlabelled EIAV peptide (the last nine amino acids of NOS1AP, DSLDDEIAV) shows no competition with F-GDLV (1 μM), with no detectable displacement at up to 300 μM (datapoints shown are mean ± SEM, *n* = 3).

This sensitivity of concerted NOS1AP binding to nNOS to denaturation has considerable potential impact on the ability of NOS1AP to compete with ligands for the nNOS PDZ pocket. To investigate this in more detail, we set up a solution-phase FP competition assay to selectively monitor the occupancy of the nNOS-PDZ ligand pocket independent of overall protein interaction, by use of a fluorescent ligand of the pocket (**Figure 2A**). Based on a previously published random peptide screen (Stricker et al., 1997) we developed the 9 amino acid FP ligand fluorescein-RRRRWGDLV, termed here ‘GDLV.’ This interacts with nNOS[1–155] with an affinity 10.7 ± 0.8 μM (**Figure 2B**), and unlabelled GDLV competes with a comparable affinity (10.7 ± 2.0 μM, **Figure 2C**). This is in sharp contrast to the C-terminal 9 amino acid PDZ ligand motif from NOS1AP, DSLDDEIAV, which was reported to have extremely low affinity (>600 μM in fluoresceinated form, Li et al., 2015; **Table 2**). Consistent with this, we cannot detect any competition for F-GDLV/nNOS[1–155] interaction by up to 300 μM unlabelled NOS1AP-9C peptide (“EIAV,” **Figure 2D**).

**Table 2:** Comparison of K_d_ values for binding of NOS1AP forms to nNOS[1–155] and Kc values for competing with F-GDLV ligand as measured by fluorescence polarization (FP).

Protein or peptide		K_d_ (overall affinity for binding nNOS[l–155] at any site(s))	Kc (affinity for nNOS-PDZ pocket, by competition fluorescence polarization)
RRRRWGDLV (synthetic)		10.7 ± 0.8 μM (FP, **Figure 2B**)	10.7 ± 2.0 μm (**Figure 2C**)
DSLDDEIAV (NOS1AP-9C)		>600 μM (FP, Li et al., 2015)	>>300 μM (**Figure 2D**)
NOS1AP 400-506 His-TAT fusion	Native	0.75 ± 0.1 μM (PD, Li et al., 2015)	0.83 ± 0.3 μM (**Figure 5A**)
	Denatured	2.4 ± 0.3 μM (PD, **Figures 1B,C**)	24 ± 2 μM (**Figure 5B**)
NOS1AP 400-503 His-TAT fusion	Native	5.8 ± 1.0 μM (PD, Li et al., 2015)	n.d.
	Denatured	5.3 ± 0.9 μM (PD, **Figures 1B,C**)	n.d.
NOS1AP 400–506 cleaved from GST		0.05 μM (Li et al., 2015)	0.52 ± 0.21 μM (**Figure 4B**)
N0S1AP 400–503 cleaved from GST		n.d.	>>10 μM (**Figure 4C**)
NOS1AP 400–506 F431A cleaved from GST		n.d.	>>10 μM (**Figure 4D**)
NOS1AP 400–503 F431A cleaved from GST		n.d.	>>10 μM (**Figure 4E**)
NOS1AP 432–506 cleaved from GST		n.d.	>>10 μM (**Figure 4F**)

*Means ± SEM are indicated. “n.d.”, several proteins cleaved from GST fusion were not measured by a comparable assay and therefore there are no K_*d*_ estimates in these cases. However by more qualitative assays NOS1AP[400-506]E429A, F431A and NOS1AP[432-506] exhibit no or reduced interaction with nNOS[1-155] (Li et al., 2015).*

### The nNOS-NOS1AP Interaction Inhibitor ZLc-002-1 Shows Weak Affinity for the nNOS PDZ Ligand-Binding Pocket

**FIGURE 3:**
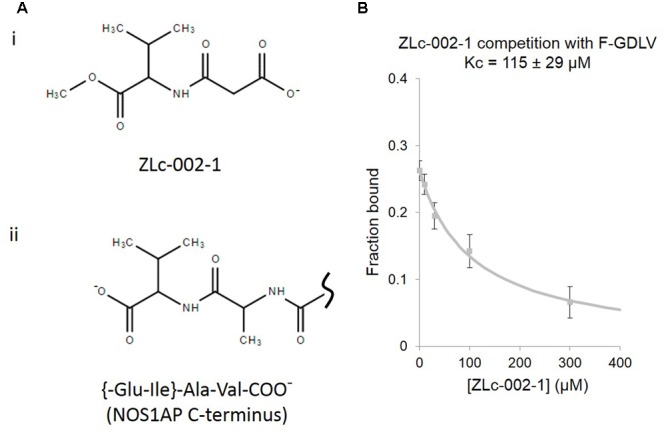
**ZLc-002-1, a prodrug-derived analog of the C-terminal PDZ ligand motif of NOS1AP, shows weak affinity for the nNOS PDZ ligand-binding pocket. (A) (i)** The structure of ZLc-002-1 [*N*-(2-carboxyacetyl)-D-valine-methyl ester], reported to be the active metabolite of the pro-drug ZLc-002 [*N*-(2-carbomethoxyacetyl)-D-valine-methyl ester], is shown. **(ii)** For direct structural comparison, the C-terminus of NOS1AP (Ala505-Val506 and preceding peptide bond only) is shown. **(B)** Competition FP assay shows the ZLc-002-1 is able to compete with F-GDLV ligand with a Kc of 115 ± 29 μM (*n* = 14).

Recently *N*-(2-carboxyacetyl)-D-valine-methyl ester, also known as ZLc-002-1, a valine-based analog of the C-terminus of NOS1AP (**Figure 3A**), has been proposed as a competitive inhibitor for nNOS PDZ pocket ligands and has shown efficacy against anxiety induced by chronic mild stress (Zhu et al., 2014). However, no actual binding or competition data has been reported for this molecule. Using the FP competition assay we find that ZLc-002-1 does compete with the PDZ pocket ligand F-GDLV (**Figure 3B**). The affinity is rather weak (>100 μM, Kc, which represents the dissociation constant of the competitor, i.e., ZLc-002-1 in this case), but it should be noted that this charged (and probably cell-impermeant) carboxylic acid species is thought to be generated in cells exposed to the cell-permeant ester prodrug ZL002 by cleavage with esterases. Ester-loading is a well-known strategy used for decades for example in loading calcium dyes into cells. In such cases, incubation with 2.5 μM uncharged ester form has been reported to lead to 200–800 μM cytoplasmic concentrations after 15 min of exposure (Hagen et al., 2012). Our observed Kc for ZLc-002-1 is therefore well within the intracellular concentration range expected from the reported 72 h incubation with 10 μM ester to obtain an effect in cell-based experiments (Zhu et al., 2014).

### The ExF Site of NOS1AP is Required to Compete With Docking of PDZ Ligand to nNOS But is Not Sufficient

**FIGURE 4:**
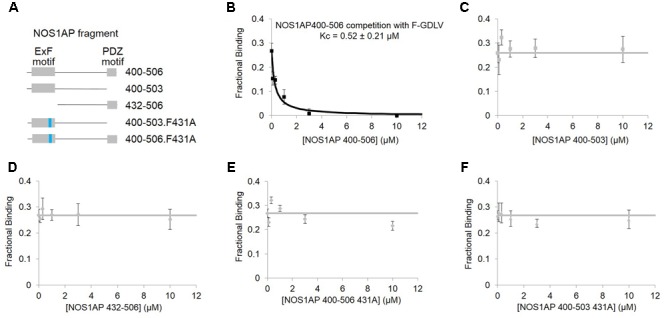
**Competition FP assay of PDZ domain pocket of nNOS shows, using purified NOS1AP sequences, that the ExF site is required to compete with docking of PDZ ligand but not sufficient. (A)** Schematic representation of different recombinant peptides cleaved and purified from GST used in this figure. **(B)** Competition FP assay shows that NOS1AP[400–506], which contains both ExF region and PDZ ligand “IAV,” competes with the PDZ pocket ligand F-GLDV with high apparent affinity (Kc = 0.52 ± 0.21 μM, *n* = 6). **(C)** Peptide NOS1AP[400–503] which binds nNOS with an affinity similar to NOS1AP[400–506] (**Figure 1B** and **Table 1**; Li et al., 2015) does not detectably exclude the PDZ pocket ligand (*n* = 6); **(D)** Similarly NOS1AP[400–506] which does exclude the ligand **(A)**, fails to do so if the ExF site is deleted (here using NOS1AP[432-506]) (*n* = 6); **(E,F)** Mutational inactivation of the ExF motif (F431A mutation) in NOS1AP[400–506] **(E)** or NOS1AP[400–503] is also sufficient to prevent any exclusion of PDZ ligand **(F)** (*n* = 6 each).

In contrast to ZLc-002-1, native NOS1AP[400–506] which contains two sites of interaction (Li et al., 2015), shows a much higher affinity in this assay (0.52 ± 0.21 μM; **Figure 4B**). NOS1AP[432–506] completely lacks the ExF motif and, like the NOS1AP-9C peptide (**Figure 2D**), also fails to compete (**Figure 4D**) even though the C-terminal PDZ ligand motif is present. Native NOS1AP[400–503], which as a His-TAT fusion binds nNOS almost as well as NOS1AP[400–506] in native state (Li et al., 2015) and equally well when denatured (**Figure 1**), does not compete with PDZ ligand in this assay (**Figure 4C**). This is the expected outcome if the ExF and PDZ ligand sites are non-overlapping and independent, because 400–503 does not have a PDZ ligand and should not be able to compete with the PDZ probe F-GDLV. Finally, NOS1AP[400–506] F431A which contains a PDZ ligand but bears an inactivated ExF motif, and NOS1AP[400–503] F431A which has neither functional ExF nor PDZ motif, each fail to compete in the FP assay (**Figures 4E,F**). In conclusion, these data show the NOS1AP PDZ ligand motif alone does not compete effectively at the PDZ ligand pocket, and nor is the functional ExF region alone sufficient. But a NOS1AP sequence containing both functional ExF region and PDZ ligand together is able to occupy the PDZ ligand pocket. This data is consistent with and provides more direct evidence than previously available in support of an earlier model of the nNOS-NOS1AP interaction (Figure 3Biii of Li et al., 2015).

### The Native State of a Structural Element in NOS1AP Greatly Increases the Affinity for the PDZ Ligand Binding Pocket of nNOS

**FIGURE 5:**
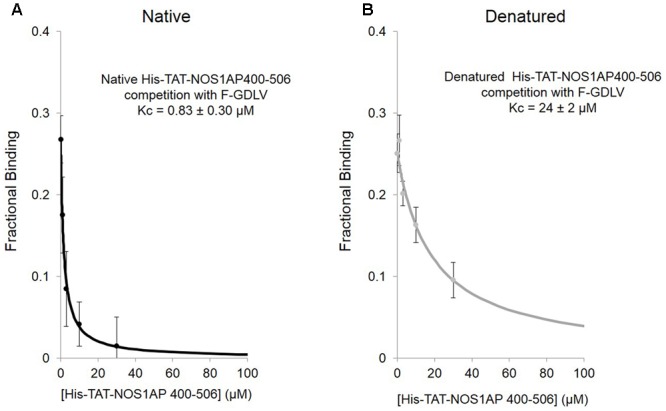
**Competition FP assay shows that the native state of a structural element in NOS1AP greatly increases the affinity for the PDZ ligand binding pocket of nNOS. (A)** Native His-tagged TAT-NOS1AP[400–506], which shows affinity of 0.75 μM by solid-phase no-wash assay (Li et al., 2015; **Table 1**) exhibits a Kc of 0.83 ± 0.30 μM for competition at the PDZ pocket, by competition FP with F-GDLV ligand (*n* = 4). **(B)** The same peptide prepared under standard denaturing conditions (as in **Figure 1**), exhibits over 30–fold lower Kc (24 ± 2 μM), a 10-fold greater reduction compared with loss of overall protein-interaction affinity seen in solid-phase assay (**Figure 1**), suggesting that the native structure required for concerted action of the ExF and PDZ ligand motifs is even more important for NOS1AP to compete at the PDZ ligand binding pocket than for binding (*n* = 4).

We applied this FP competition assay to the native and denatured His-TAT-fused NOS1AP[400–506] peptides that we had already characterized by the solid phase assay (**Figure 1**; Li et al., 2015). We find that native His-TAT- NOS1AP[400–506] competes with F-GDLV, whereas denatured His-TAT-NOS1AP[400–506] interaction is 30-fold weaker (**Figures 5A,B**; significant difference at *P* < 0.0001, two-tailed unpaired *t*-test, *n* = 4). This difference greatly exceeds the ∼3-fold impact of denaturation on overall binding affinity by no-wash pulldown assay (**Figure 1**; K_d_ and Kc data are compared in **Table 2**). In the competition-FP assay we only monitor the state of the PDZ ligand pocket so this data alone does not distinguish between denaturation destroying the affinity of the ExF region or disrupting the concerted action of the ExF region and PDZ motif. However, considering the protein-interaction results shown in **Figure 1** and **Table 1** (also Li et al., 2015), we can conclude that the interaction by the ExF region is resistant to denaturation and it is the concerted action of the two sites to occlude the pocket that fails upon denaturation. Furthermore it is clear that the denaturation procedure used here does not merely result in loss of functional protein by aggregation as this would affect all binding events similarly but the data shows a 30-fold effect on PDZ ligand occupancy and no effect on nNOS binding the via ExF motif region (compare Kc for NOS1AP[400–506] with and without denaturation and K_d_ for NOS1AP[400–503] with and without denaturation, **Table 2**).

### Molecular Dynamics Simulation of nNOS-NOS1AP PDZ Ligand Interaction

Two possible explanations are that either the PDZ motif itself is denaturable or that there is an additional denaturable element required for facilitating the concerted action of the two interaction sites. The term “denaturable” refers here to an element the properties of which are lost after denaturation of its structure, and excludes intrinsically disordered sequences. This does not necessarily imply that denaturability is itself a functional property that is modulated *in vivo* although in some cases this has been demonstrated (Mitrea and Kriwacki, 2013; Csizmok et al., 2016).

Canonical C-terminal PDZ ligands are short peptides, typically too short to have a stable structure, and are thus considered to represent a class of intrinsically disordered regions (Ivarsson, 2012). The NOS1AP C-terminal PDZ ligand itself, residues 504–506 and potentially those preceding it, is therefore not by itself expected to form a stable structure. Any stable structure it might adopt in free solution could lead to an energy barrier impeding the acquisition of the bound state in the PDZ ligand binding pocket. We wished to more precisely predict how this might influence the interpretation of the binding data. Therefore, we carried out a molecular dynamic simulation based on the existing NMR structure for nNOS docked with the melatonin receptor peptide (1B8Q.pdb). This process calculates the positions of all atoms from a starting point after each of a number of specified small time steps. This models a relevant atomic-level representation of the binding event between the peptide and protein in the predicted binding pocket and reveals the biophysics of this binding event. This can provide support for a specific hypothesis or it can contradict it. For example if the simulation shows a candidate ligand leaves a binding pocket, the binding event is predicted to be non-existent.

**FIGURE 6:**
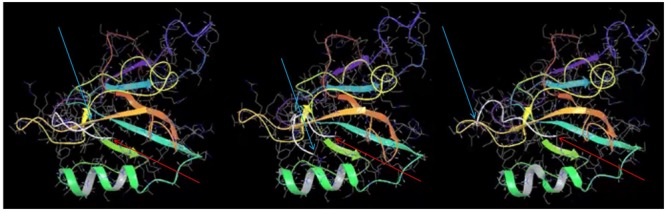
**Molecular dynamics simulation of nNOS-NOS1AP PDZ ligand interaction suggests only the last three C-terminal residues of NOS1AP reside in the ligand binding pocket.** Three frames at 0.15 ns intervals are shown from a molecular dynamics simulation of the NOS1AP C-terminus (last nine amino acids, C-alpha chain is represented as a *white* line) docked in the PDZ pocket of the nNOS-extended PDZ domain. Note one end (C-terminal valine indicated with thin red arrow) remains close the PDZ-β2 (orange), while the N-terminal end (N-terminal indicated with thin cyan arrow) moves rapidly in the nearby space but is not positioned over any one surface. The simulation (full 10 ns movie file in the Supplementary Movie) suggests only the 3 C-terminal residues within the 9-mer ligand form stable interactions during this simulation.

Comparing the docking of MelA peptide in the published NMR structure (Tochio et al., 1999) and NOS1AP PDZ ligand (**Figure 6** and Supplementary Movie) reveals that interaction of the peptides with the PDZ pocket is predominantly based on backbone–backbone interactions rather than any specific rigid conformation of side chains. Specifically, the hydrogen bond interactions between the peptide and protein backbones are the key driving force of the interaction rather than other forces, (i.e., hydrophobicity, van der Waals forces). The simulation (**Figure 6** and Supplementary Movie) specifically shows that the last three residues remain docked in the pocket whereas the remaining six residues move rapidly over the protein on a picosecond timescale and do not remain docked with any specific surface region of nNOS. The results of the simulation are therefore consistent with (i) a lack of requirement for a pre-formed structure of the PDZ ligand motif for binding to the PDZ pocket and (ii) a participation of only the 3 most C-terminal amino acids of the ligand (isoleucine-alanine-valine) in the interaction with nNOS.

We conclude that a pre-existing structure of the PDZ ligand in NOS1AP is unlikely to be required. For these reasons the disruption of the concerted action of the two binding sites by denaturation of the NOS1AP C-terminal nNOS binding domain cannot be ascribed to either motif alone - neither the internal ExF motif, as it interacts even if denatured (**Figure 1**), nor the PDZ ligand as it interacts without adopting a specific fold (**Figure 6**).

### Secondary Structure Prediction of the C-Terminal nNOS Binding Region of NOS1AP

**FIGURE 7:**
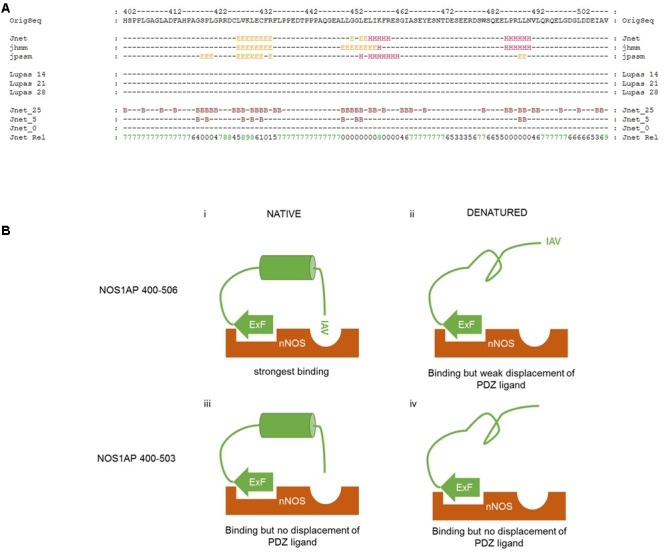
**Proposed interplay between different regions of NOS1AP on interact with the N-terminal region of nNOS. (A)** Secondary structure prediction applied to the C-terminal region of NOS1AP (the human sequence is shown). The prediction shown here generated by the JPRED3 engine aligns multiple prediction algorithms – the Jnet secondary structure prediction and residue burial prediction (0, 5, and 25% exposure to solvent for Jnet_0, _5 and _25), HMMER hidden markov model profile-based prediction (jhhm) and PSI-BLAST position-specific scoring matrix prediction (jpssm) as well as Lupas coil predictions at window sizes 14, 21, and 28 as indicated (“-“ for each residue indicates less than 50% probability). Jnet Rel indicates reliability of prediction from 0 (lowest) to 9 (highest). Consensus predictions indicate three regions of secondary structure and residue burial around (i) a predicted β-sheet region in the vicinity of the previously described ExF motif, (ii) a helix prediction between residues 452-460 and (iii) a predicted α-helix from residues 484–489. **(B)** Schematic representation of the interaction of different states of the C-terminal nNOS binding region of NOS1AP with the N-terminal PDZ-domain containing region of nNOS, indicating the state of occlusion of the PDZ ligand-binding pocket in each case. **(i)** Native NOS1AP 400–506 interacts via the ExF site with nNOS, and via the C-terminal IAV motif with the PDZ ligand binding pocket of nNOS as shown in the simulation of **Figure 6**. This is sterically possible and/or favored because of an intervening possibly α-helical structure positioning both motifs appropriately, and the pocket is occupied, excluding other ligands (such as F-GDLV in the FP assays); **(ii)** Denatured NOS1AP 400–506 loses the putative α-helical structure, can still interact via the ExF motif region, but the IAV sequence alone has too low an affinity and is not able to interact with nNOS at the same time as the ExF motif as a result of the loss of intervening structure. The pocket is free to bind other ligands; **(iii)** NOS1AP 400–503 similarly can interact with nNOS via the ExF motif but has no IAV PDZ ligand. Thus, even though structural integrity of the intervening sequence may be preserved, overall interaction is not enhanced by any C-terminal interaction and importantly, this sequence is therefore unable to compete with ligands interacting at the PDZ binding pocket. **(iv)** Denaturation of NOS1AP 400–503, though it disrupts the structural integrity of the intervening sequence, has little consequence to interaction because the ExF motif region is resistant to denaturation and no other region contributes when the IAV sequence is deleted. The pocket is free to bind other ligands.

We have found that denaturation does not affect the affinity of the ExF-containing region of NOS1AP for nNOS (residues 400–503, **Table 1**). MD simulation suggests the C-terminal PDZ motif interaction involves only 3 amino acids (**Figure 6**), which is too short to be sensitive to denaturation. Therefore, the denaturation-induced sharp decline in competition at the PDZ pocket (**Figure 5** and **Table 2**) as well as the reduced interaction with nNOS of denatured NOS1AP containing both these sites (residues 400–506, **Table 1**) indicates that denaturation targets a third, previously unidentified, element in the C-terminal region of NOS1AP (residues 400–506) that is required for optimal binding to nNOS. We specifically infer a folded structural element most likely residing between the ExF region and the PDZ ligand at the extreme C-terminus. As the PDZ ligand of NOS1AP interacts with the PDZ domain pocket and deletion of only three amino acids from the C-terminus of NOS1AP eliminates occlusion of the PDZ ligand binding pocket (**Figure 4C**), the ExF motif region must interact at another site on the compact nNOS N-terminal domain. The concerted simultaneous binding of both sites to the nNOS domain must impose steric requirements on their relative orientations, as was previously demonstrated for efficient binding of dimeric ligands to the tandem PDZ domains of PSD95-PDZ1-2 (Long et al., 2003). In the case of nNOS-NOS1AP interaction it seems likely that the steric requirements could be fulfilled by the inferred folded structural element of NOS1AP. To explore the possible presence of folded elements, we applied secondary structure prediction methods to the C-terminal nNOS binding region, i.e., NOS1AP residues 400–506 (**Figure 7A**).

The JPRED3 engine (Cole et al., 2008) predicts two main structural elements within the C-terminal nNOS binding region. The first is a β-sheet region around the ExF motif, from residues 425 to 432. The requirements for residues 429 and 431 but not 430 is consistent with a beta sheet structure bearing residues with alternating orientations, but the equal affinities of denatured 400–503 and 400–506 (**Figure 1**) suggests the ExF motif readily adopts a binding conformation even after denaturing conditions. The second structural element is a putative α-helix from 452 to 460, with a third predicted, again α-helical, around residues 484–489. These putative helical regions may potentially represent a denaturable element that could serve a role in orientation of ExF and PDZ ligand motifs and conversely denaturing them might then lead to steric incompatibility of the two sites to bind a single nNOS molecule, which is what we observe.

## Discussion

Our data show that not only are two binding elements, the ExF motif and PDZ ligand required for the optimal binding of NOS1AP to nNOS as described before (Li et al., 2015), but that there is in addition a requirement for a denaturable structural element for the concerted action of these two sites (**Figures 7Bi,ii**). This is presumably important to provide appropriate steric conditions for the two sites to simultaneously access their respective binding sites on nNOS. Although it was previously shown (Li et al., 2015; and shown again here in **Figures 1**, **4**, **5**) that the NOS1AP C-terminal PDZ ligand motif was dispensable for binding of NOS1AP to nNOS – the ExF motif region was sufficient for binding – it was not previously clear whether or not the ExF-interaction also occluded the PDZ motif site of the bound nNOS molecule by either steric or allosteric mechanisms, or left it vacant and able to interact. Here we have demonstrated directly that the interaction of NOS1AP to nNOS via the ExF motif region and PDZ ligand interaction are separable events, and thus binding of nNOS by NOS1AP[400–503] via the ExF motif alone has no detectable occluding effect on the PDZ pocket which remains equally able to bind ligand (shown schematically in **Figure 7Biii**; corresponding data in **Figure 4C**).

Conversely, we provide here an estimate of the apparent competitor affinity *at the PDZ ligand binding pocket* by a NOS1AP sequence containing the C-terminal PDZ ligand motif. The latter motif of NOS1AP in isolation is known to have extremely weak affinity for nNOS (**Figure 2**; Li et al., 2015; **Table 2**). It is also known that the presence of this ligand together with the ExF motif within the C-terminal 107 amino acids of NOS1AP, (i.e., residues 400–506) generates a high affinity interaction with nNOS as previously described (Li et al., 2015) but this does not mean the PDZ pocket would be occupied with the same apparent affinity as the overall dissociation constant for the NOS1AP-nNOS complex. We show directly that the two motifs together lead to a high affinity occupancy of the PDZ ligand binding pocket (apparent Kc ∼0.5 μM, **Figure 4B**) that efficiently competes with an alternate model ligand (F-GDLV). It is this efficient competition at the pocket that requires three components – the ExF motif, the C-terminal PDZ ligand and native conformation of a denaturable element within residues 400-506 of NOS1AP (**Figure 7Bi**) - not the overall NOS1AP-nNOS interaction for which the ExF motif site is sufficient (e.g., **Figure 7Biii**). Thus, without a native structure, the ExF motif-containing region still interacts with nNOS (**Figure 1** and **Table 1**) but the contribution of the PDZ ligand, which is believed to be critical for signaling (Li et al., 2013, 2015), is lost (**Figure 7Bii**; cf. **Figure 5B**). The impact in terms of competition at the PDZ pocket is similar to deletion of the C-terminal PDZ ligand (**Figures 7Biii,iv**; cf. **Figure 4C** and **Table 2**).

We previously reported that it is possible to pharmacologically inhibit the cellular impact of nNOS-NOS1AP interaction using a competing PDZ ligand (Li et al., 2013) and more recently reported a complementary approach using the ExF motif bearing peptide devoid of PDZ ligand (Li et al., 2015). The distinct third requirement for a structural component that we report here, is potentially a source of additional inhibitors. Structure-based drug design may not easily target this element, particularly in the complete absence of structural coordinates of the molecule. The precise location of the third element is not known, but it is likely to reside between ExF motif (residues 429–431) and PDZ ligand (residues 504–506). The predicted alpha-helical regions between residues 452–460 and/or 484–489 are therefore potential candidates. Even while the structure of NOS1AP remains unsolved, the contributions of these two candidate regions could nevertheless be evaluated by mutational approaches. However, even without actually locating the residues making up this denaturable element, future library screens could reveal HTS hits that act at this site. It is not uncommon for the actual binding sites of HTS-derived protein–protein interaction inhibitors to be unclear. For example, IC87201 inhibits the interaction of nNOS with PSD95 and it is believed to interact with nNOS (Florio et al., 2009) but that actual site of interaction of the nNOS molecule has not been mapped. A similar molecule, ZL006, was in fact described as having been designed precisely to disrupt the interaction between two secondary structure elements (Lai and Wang, 2010; Zhou et al., 2010), though once again the actual interaction site has yet to be determined (Bach et al., 2015). Therefore, it is important to identify the precise requirements for protein–protein interaction, not only for the design of new inhibitor molecules but also to interpret and fully understand the mechanism of inhibition by HTS hits so that their properties can be rationally explained and hits can be optimized. For instance, a screen for modulators targeting the denaturable element we have reported here would most easily be addressed using an assay that reports its function, i.e., allowing the concerted action of PDZ ligand and ExF motif for interaction of NOS1AP binding to nNOS. Such a screen would therefore inevitably also report inhibitors of both the PDZ ligand interaction and the ExF motif interaction. The actual site of action of screen hits would therefore have to be deconvolved by additional site-specific assays once screen hits have been revealed and the knowledge that three distinct elements are involved in the interaction is required to successfully achieve this.

We previously demonstrated that the PDZ ligand contributes an increased stability of the interacting complex and that competition at the PDZ ligand site alone inhibits signaling, presumably due to a reduced lifetime of this complex impacting on NMDA receptor/nNOS-dependent signaling outputs. The additive impact of the PDZ ligand on affinity is shown here to be largely lost after denaturation. This suggests that any successful targeting of the novel denaturable structural element revealed in this report would likely reduce the affinity of NOS1AP for nNOS and lifetime of the nNOS-NOS1AP complex in a manner similar to that we have observed by competition at the PDZ ligand-binding site (Li et al., 2015). Structural elements in proteins are not completely rigid and there is no evidence that the C-terminal region of NOS1AP acts like a highly stable globular protein. Therefore, it is reasonable to expect that the required structural element has sufficient dynamic ‘breathing’ movement to allow its targeting by small molecules in analogous way to that proposed for ZL006 (Lai and Wang, 2010; Zhou et al., 2010). Moreover, given the requirement for this element for optimal interaction of NOS1AP with nNOS and for occlusion of the PDZ ligand-binding pocket, any dynamic reorganization of this element could potentially form the basis of additional cellular regulation of nNOS-NOS1AP interaction. However, such cellular regulation is speculative as there is currently no evidence to support an endogenous regulation of this element at this time. Nevertheless, it should be noted that NOS1AP is not the only protein reported to interact with the PDZ ligand-binding pocket of nNOS. Other candidates for interaction at this site include phosphofructokinase-M (Firestein and Bredt, 1999), the melatonin A receptor (MelA, Tochio et al., 1999), alpha-adrenergic receptors (Pupo and Minneman, 2002), the transcriptional corepressor carboxyl-terminal binding protein 1 (CtBP1, Lin et al., 2003), and DHHC domain palmitoyl acetyl transferases such as ZDHHC23/NIDD (Saitoh et al., 2004). Therefore, the ability of NOS1AP to occlude the nNOS PDZ-binding pocket, the requirement of the NOS1AP ExF motif and additional structural element in this occlusion and the possible pharmacological manipulation or even endogenous regulation of these determinants would be expected to determine whether nNOS is competent to recruit these additional candidate interacting proteins. This in turn could reshape nNOS-dependent signaling profiles and thus influence disease mechanisms involving nNOS.

## Conclusion

Our findings extend our understanding of nNOS-NOS1AP interaction by revealing the contribution of three separate elements. In addition to (i) the recently described internal NOS1AP-ExF motif region which is sufficient for interaction (Li et al., 2015), and (ii) the initially described NOS1AP-PDZ ligand which is not sufficient alone but increases stability and affinity in the presence of the ExF motif (Jaffrey et al., 1998; Li et al., 2015), we provide evidence here for (iii) a third NOS1AP element, the structure and therefore function of which is sensitive to denaturation, that orients the other two NOS1AP elements to allow them to form a stable interaction with nNOS and occlude alternate ligands from the nNOS ligand binding pocket. It would be important to determine if this third element is merely encoded in a robust and static manner by the amino acid sequence of NOS1AP or whether it is dynamically regulated by cellular signaling pathways and whether it is amenable to pharmacological modulation. Our findings therefore have implications for drug discovery opportunities for the numerous diseases that have been linked to nNOS and NOS1AP as well as the manner in which the small molecule inhibitor compounds for the different sites could be identified. It is important to consider that NOS1AP has been reported to mediate the activation of p38MAPK (Li et al., 2013, 2015), a kinase which is in turn linked to excitotoxicity (Cao et al., 2004, 2005; Semenova et al., 2007), neurodegeneration (Dau et al., 2014; Ittner et al., 2014; Roy et al., 2015), neuropsychiatric conditions (Bruchas et al., 2011; Ehrich et al., 2015) and neuropathic pain (Berta et al., 2016). As the neurological side effects of direct p38MAPK inhibitors have been addressed in clinical trials by reducing brain penetrance (Krementsov et al., 2013), inhibiting p38MAPK in the CNS may be difficult. Targeting disease-related upstream mechanisms such as NOS1AP could in this case be an attractive alternative approach. Moreover, as NOS1AP has itself been directly associated with a range of disorders and diseases, it would be valuable to develop mathematical models to describe and predict its function and regulation under different conditions. As more details emerge of the regulatory mechanisms of NOS1AP, such as those we present here, a model to predict the interaction of NOS1AP with its targets and the impact of inhibitors at specific sites on these interaction surfaces becomes increasingly feasible.

## Author Contributions

L-LL carried out the experimental work, its analysis, interpretation and presentation, acquired necessary resources and participated in writing the manuscript. KC carried out, analyzed and described the molecular dynamics simulation. MC planned and supervised the project, participated in the analysis, interpretation and presentation of the data, acquired and provided the resources required and wrote the manuscript.

## Conflict of Interest Statement

The authors declare that the research was conducted in the absence of any commercial or financial relationships that could be construed as a potential conflict of interest.
